# New perspective on glycoside hydrolase binding to lignin from pretreated corn stover

**DOI:** 10.1186/s13068-015-0397-6

**Published:** 2015-12-18

**Authors:** John M. Yarbrough, Ashutosh Mittal, Elisabeth Mansfield, Larry E. Taylor, Sarah E. Hobdey, Deanne W. Sammond, Yannick J. Bomble, Michael F. Crowley, Stephen R. Decker, Michael E. Himmel, Todd B. Vinzant

**Affiliations:** Biosciences Center, National Renewable Energy Laboratory (NREL), 15013 Denver West Parkway, Golden, CO 80401 USA; Applied Chemicals and Materials Division, NIST, Boulder, CO 80305 USA; Department of Veterans Affairs, Boise, ID 83702 USA

**Keywords:** Lignin, Glycoside hydrolase, Enzyme binding, Cellulase, Biomass, Pretreatment

## Abstract

**Background:**

Non-specific binding of cellulases to lignin has been implicated as a major factor in the loss of cellulase activity during biomass conversion to sugars. It is believed that this binding may strongly impact process economics through loss of enzyme activities during hydrolysis and enzyme recycling scenarios. The current model suggests glycoside hydrolase activities are lost though non-specific/non-productive binding of carbohydrate-binding domains to lignin, limiting catalytic site access to the carbohydrate components of the cell wall.

**Results:**

In this study, we have compared component enzyme affinities of a commercial *Trichoderma reesei* cellulase formulation, Cellic CTec2, towards extracted corn stover lignin using sodium dodecyl sulfate-polyacrylamide gel electrophoresis and *p*-nitrophenyl substrate activities to monitor component binding, activity loss, and total protein binding. Protein binding was strongly affected by pH and ionic strength. β-d-glucosidases and xylanases, which do not have carbohydrate-binding modules (CBMs) and are basic proteins, demonstrated the strongest binding at low ionic strength, suggesting that CBMs are not the dominant factor in enzyme adsorption to lignin. Despite strong adsorption to insoluble lignin, β-d-glucosidase and xylanase activities remained high, with process yields decreasing only 4–15 % depending on lignin concentration.

**Conclusion:**

We propose that specific enzyme adsorption to lignin from a mixture of biomass-hydrolyzing enzymes is a competitive affinity where β-d-glucosidases and xylanases can displace CBM interactions with lignin. Process parameters, such as temperature, pH, and salt concentration influence the individual enzymes’ affinity for lignin, and both hydrophobic and electrostatic interactions are responsible for this binding phenomenon. Moreover, our results suggest that concern regarding loss of critical cell wall degrading enzymes to lignin adsorption may be unwarranted when complex enzyme mixtures are used to digest biomass.

## Background

It has been well established that overall lignin content and its localized distribution post-pretreatment is inversely proportional to the enzymatic hydrolysis of the remaining carbohydrate content [1–4]. At elevated pretreatment temperatures (>140 °C), lignin is above its glass transition phase and separates from the carbohydrate polymers of the cell wall, presumably driven by lignin–lignin hydrophobic affinity following transition from solid to liquid. Microscopic droplets of various sizes of lignin appear on cell wall surfaces after thermochemical pretreatment, as verified by antibody and energy-dispersive X-ray spectroscopy (EDAX) analysis [5]. Researchers have also shown a clear correlation between thermochemical pretreatment severities and the size and number of surface lignin droplets [5]. It appears that the presence of these droplets may decrease the rate of enzymatic saccharification, either by physically blocking access of cellulases to cellulose microfibrils or by increasing the non-productive adsorption of enzymes [5].

For decades, pretreatment research has focused on chemistries and processes which selectively or effectively remove hemicellulose and lignin from the plant cell wall to increase enzyme accessibility to and subsequent hydrolysis of cellulose to glucose. Although significant advances have been made in pretreatment processes to improve the accessibility of enzymes to cellulose, efficient and high-yield hydrolysis still requires large quantities of enzymes [6, 7]. Reducing this loading (and associated cost) can be approached by (1) increasing the specific activities of the major components of the cellulase complex, (2) engineering the ratios and types of activities expressed in industrial fungal systems to work more synergistically and effectively with specific biomass feedstock’s and pretreatment chemistries, and (3) employing various enzyme recycle strategies. Regardless of the approach(es) used, lignin remains a major factor in biomass recalcitrance.

Several issues remain regarding the presence of lignin. Complete removal of lignin results in an overall increase in cellulose crystallinity, which corresponds to a reduction in its digestibility. We note that in an industrial context, complete lignin removal is also cost prohibitive for biofuel production [8, 9]. While advantageous to the pulping industry, such a process would not be optimal for the biofuel industry. Nakagame et al. determined the major variables in non-productive adsorption of hydrolytic enzymes to lignin [10–12]. They also showed that lignin from different plant origins coupled with varied pretreatment chemistries and severities may result in a variable adsorption surface chemistry and enzyme accessibility [11, 12]. They developed potential pretreatment strategies that may alter lignin surface chemistries to effectively keep enzymes from adsorbing to the surface of lignin under process-relevant pH and ionic strength conditions. Other groups have worked on modifying the surface chemistry of lignin to reduce its affinity toward enzymes. Lou et al. used sulfite pretreatment revealing that pH-induced lignin surface modification reduces the nonspecific cellulase binding to lignin and enhances enzymatic saccharification at elevated pH (i.e., pH 5.5 and higher) [13].

**Fig. 1:**
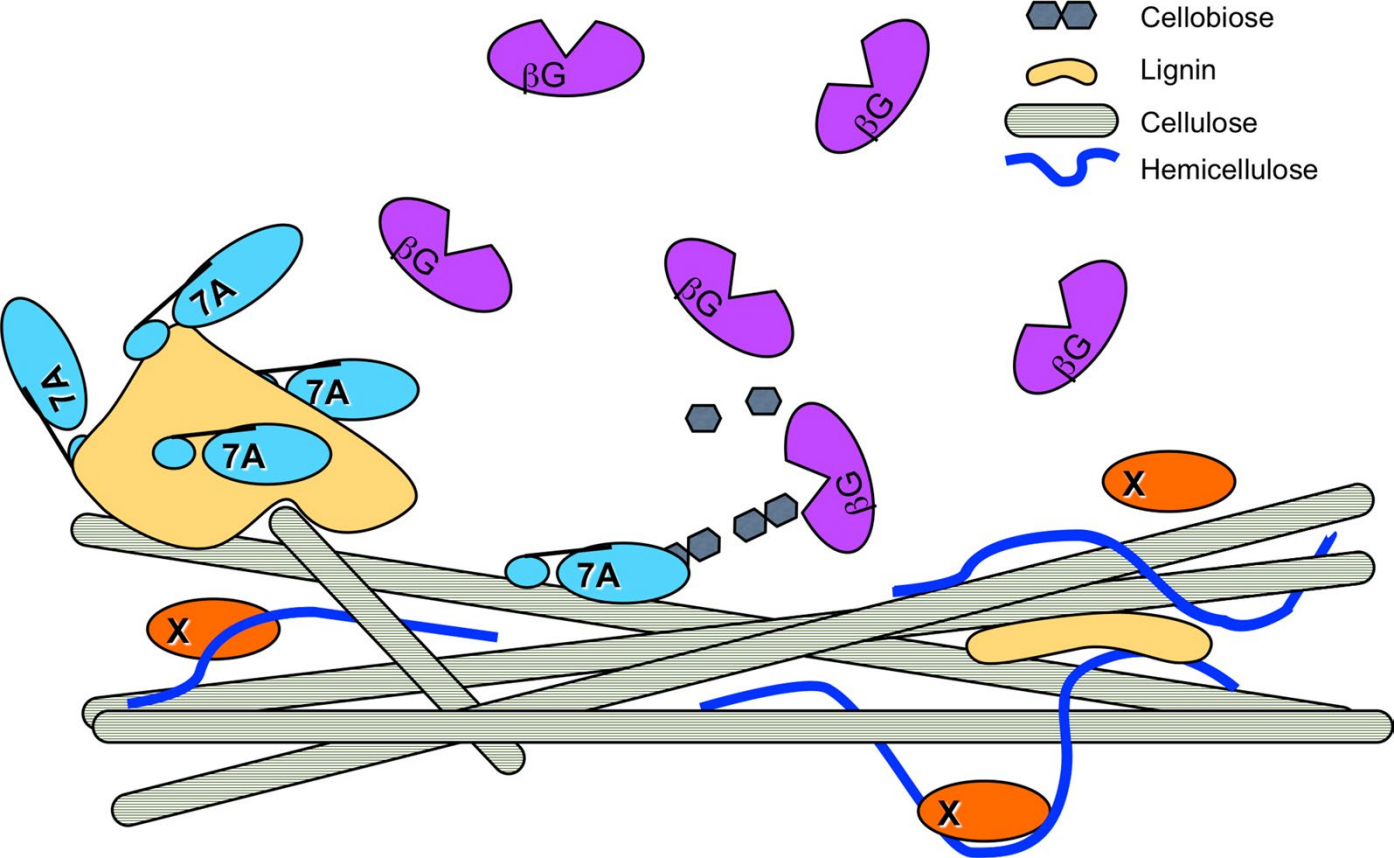
Current paradigm of carbohydrate binding modules (CBMs) having the highest affinity toward lignin. Here Cel7A with a CBM adsorbs to lignin and is sequestered away from the cellulose, rendering it ineffective. Other enzymes without CBMs do not adsorb to lignin

Many evolving hypotheses and approaches are being explored to gain a better understanding of the nature of non-productive adsorption of cellulases and hemicellulases to the lignin surface. It is generally accepted that enzyme–lignin interactions are non-covalent in nature and are likely due to hydrophobic and electrostatic interactions; as well as possibly hydrogen bonding and charge transfer effects, such as pi-orbital electron interactions [14]. Several research groups have focused on individual enzyme types and families to measure specific enzyme adsorption rates to lignin and to elucidate the lignin-binding mechanism. This approach; however, does not measure how these enzymes interact with lignin in a mixed population of hydrolytic proteins. These studies have led to the current paradigm that carbohydrate-binding domains, with hydrophobic residues positioned towards the substrate surface, cause non-productive adsorption (Fig. 1) [15, 16]. Palonen et al. demonstrated using steam-pretreated softwood that Cel7A and its catalytic domain exhibited higher affinity to the softwood in comparison to Cel5A and that removing the carbohydrate-binding module (CBM) for Cel7A led to a significant decrease in its binding efficiency [15]. Similar results were reported by Rahikainen et al. using lignin isolated from steam explosion pretreated and non-pretreated spruce and wheat straw and measured using a quartz crystal microbalance with dissipation. They demonstrated an increase in the binding efficiency with Cel7A fully intact (both the catalytic domain and the CBM) in comparison to only the catalytic domain of Cel7A [16]. Others have suggested that irreversible adsorption can be caused by exposed hydrophobic core amino acids binding to lignin during heat-induced denaturation [17]. An alternative theory suggests lignin-induced deactivation is due to the non-specific binding of small phenylpropane units derived from low-molecular-weight lignin (LMWL) which acts as inhibitors and functionally blocking enzyme active sites, rendering non-bound enzymes inactive [18, 19].

**Fig. 2:**
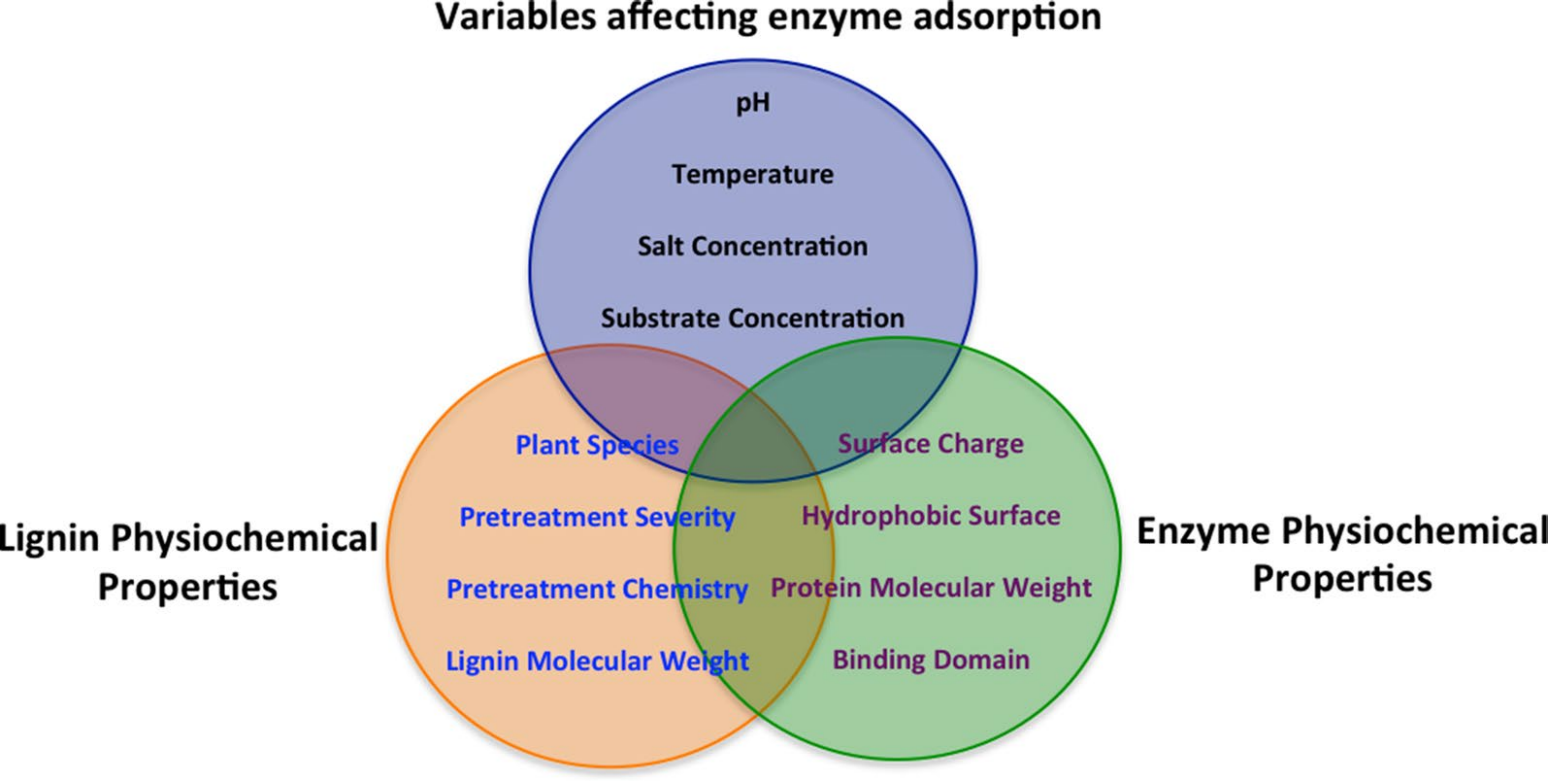
Lignin–cellulase relationships between physiochemical properties of proteins and substrates; as well as the parameters of the biomass conversion process are shown

Aside from inhibition by LMWL, the interaction between lignin and enzymes is dependent upon multiple factors based on properties of the proteins and lignins. As protein properties vary significantly across and even within enzyme families, a simple direct relationship is not obvious. This relationship is also compounded by varying lignin structural and chemical properties inherent in the lignin source (i.e., feedstock plants) and altered by pretreatment chemistry and severity. This lignin–cellulase interaction paradigm describes the relationship between the physiochemical properties of both the protein and the pretreated biomass substrate as shown in Fig. 2.

Advances in pretreatment technologies and commercial enzyme preparations have routinely provided lignocellulosic biomass conversion yields of 85–90 %, yet the dogma persists that lignin adsorbs hydrolytic enzymes through non-specific hydrophobic interactions. We proposed to determine which specific enzymes in a complex cellulase mix are affected by the presence of steam explosion at 180 °C pretreated corn stover lignins and to evaluate what overall mechanisms and controllable process parameters have the strongest influence on adsorption. An enzyme’s affinity for a specific substrate can be understood by considering its inherent physiochemical properties, such as molecular weight, surface amino acid charges and hydrophobicity, and the capacity for inter-molecular interactions, such as hydrogen bonding or pi-orbital effects. These factors affect interactions between individual enzymes, as well as enzyme interaction with lignin and other biomass components. All these interactions occur in a competitive way, following the principle of the Vroman Effect, until an equilibration state driven by the enzymes’ structure and affinity for substrates has been reached. Many research groups focus on individual enzymes or enzyme types and demonstrate adsorption rates and mechanisms that may not be displayed in a mixed population of proteins, where the competitive binding shown by multiple enzymes for the same substrate(s) may affect enzyme–substrate interactions [16, 17, 20]. This work explores how a mixed population of enzymes typically found in commercial cellulases interacts with lignin and if these interactions differ compared to those of individual purified enzymes.

## Results and discussion

### Which enzymes are affected?

Commercial cellulases fall into several broad groups, such as native, single-strain secretomes, multiple-strain mixed secretomes, and engineered-strain secretomes containing one or more non-native activities introduced to enhance a particular activity. To determine if hydrolytic enzymes are adsorbing to the surface of lignin and if this binding affects the secretome activity, tractable binding/activity experiments were designed using Cellic CTec2 commercial cellulase and lignin extracted from pretreated corn stover. Individual enzymes were tracked by mixing CTec2 with lignin and analyzing proteins partitioned into the bound (lignin pellet) and unbound (supernatant) fractions. A range of *para*-nitrophenol (*p*NP) substrates, including *p*NP-β-d-lactopyranoside (*p*NPL), *p*NP-β-d-cellobioside (*p*NPC), *p*NP-β-d-xylopyranoside (*p*NPX), and *p*NP-β-d-glucopyranoside (*p*NPG) were used to estimate the type of activity remaining in each fraction.

**Fig. 3:**
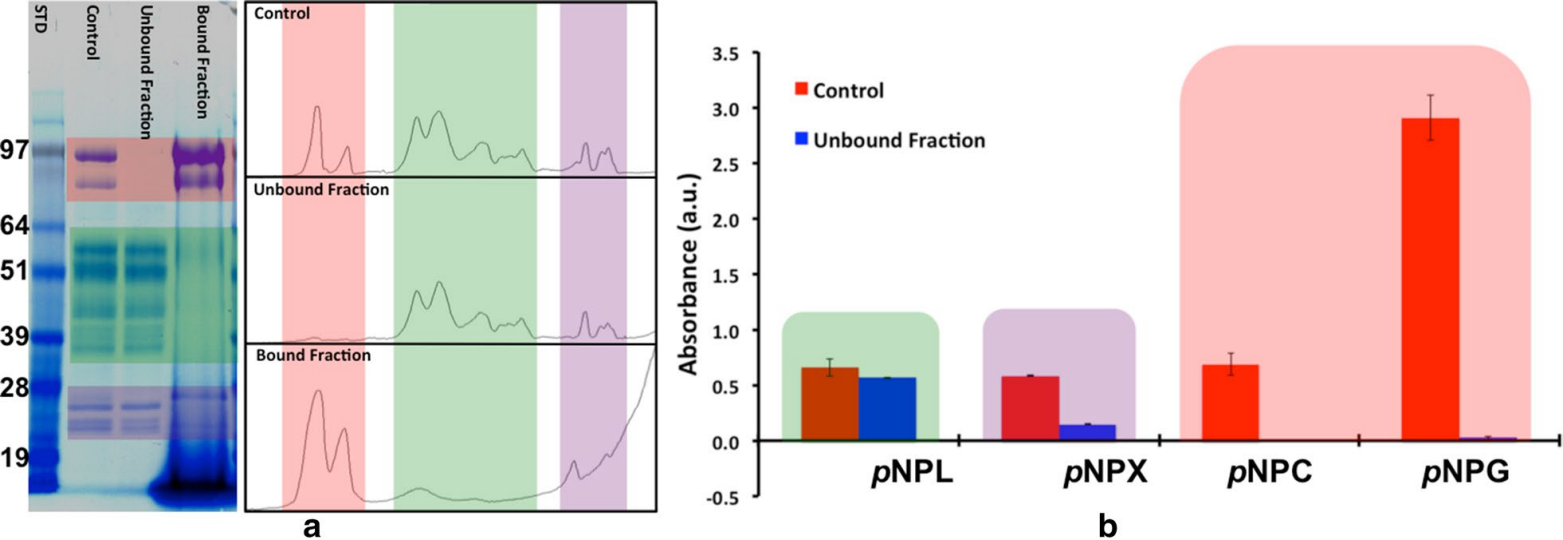
**a** SDS-PAGE gel comparing the supernatant and lignin pellet from CTec2 with the different molecular regions highlighted (*rose* = high MW, *green* = mid MW, *purple* = low MW). The gel has the following lane assignment: *1* Molecular weight standard, *2* CTec2 control, *3* Unbound fraction, and *4* Bound fraction. **b** Bar graph showing the activity of CTec2 on the different *p*NP-substrates

After incubating CTec2 with lignin for 1 h, the mixtures were centrifuged and proteins in the supernatants (unbound) and pellets (bound) were visualized by gel electrophoresis (Fig. 3a). Intensity plots of the gel lanes were generated using the ImageJ Gel analysis application plugin. This tool allows for the visualization of the intensity and the overall shape of the protein bands. Differential banding patterns between bound, unbound, and control (CTec2 only, no lignin) were used to determine which bands in a mixed population of enzymes had the highest affinity for lignin. The *p*NP activities were used similarly to track where the different activity types partitioned. For assays of the supernatants, the difference between activity levels in CTec2 control and the unbound fraction was used to estimate the percentage of bound activity in each fraction.

Individual proteins in CTec2 bind differently to lignin. Controls of protein- and lignin-only samples indicate the proteins can be found in either the bound or unbound fractions (Fig. 3a). The high-molecular-weight proteins (rose highlights, Fig. 3a) and low-molecular-weight proteins (purple highlights, Fig. 3a) have a high affinity for lignin, as they are absent in the unbound fractions and prominent in the bound fractions. Intermediate-molecular-weight proteins (green highlight, Fig. 3a) were largely unbound. Note that the large band located at the bottom of the bound fraction is the lignin pellet. Figure 3b shows the activity profile of CTec2 (red bars) and unbound fraction (blue bars). We conclude that the combination of activity loss and bound molecular weights can be used for identifying the proteins binding to lignin. Major bound activities include *p*NPC and *p*NPG, presumably attributable to β-d-glucosidase enzymes present in the high-molecular-weight (>80 kDa) bands. The *p*NPX activity was also mostly bound to lignin and is presumably correlated with the bound low-molecular-weight (<30 kDa) proteins, considering that xylanases are typically of low-molecular-weight. Cellulase activity, indicated by the *p*NPL activity, remained mainly unbound and is presumed to be associated with the intermediate-molecular-weight (30–80 kDa) bands. Table 1 shows the overall percent of *p*NP activity bound to lignin.

**Table 1:** Percent *p*NP activity lost due to enzymes bound to lignin

*p*NP substrate	% Activity loss in *p*NP in the unbound fraction
*p*NP-lactopyranoside (cellobiohydrolases, cellulases)	14
*p*NP-xylopyranoside (xylanases; β-d-xylobiosidases)	75
*p*NP-cellobioside (β-d-glucosidases, endoglucanases)	100
*p*NP-glucopyranoside (β-d-glucosidases)	99

### What are the effects of protein adsorption to lignin?

**Fig. 4:**
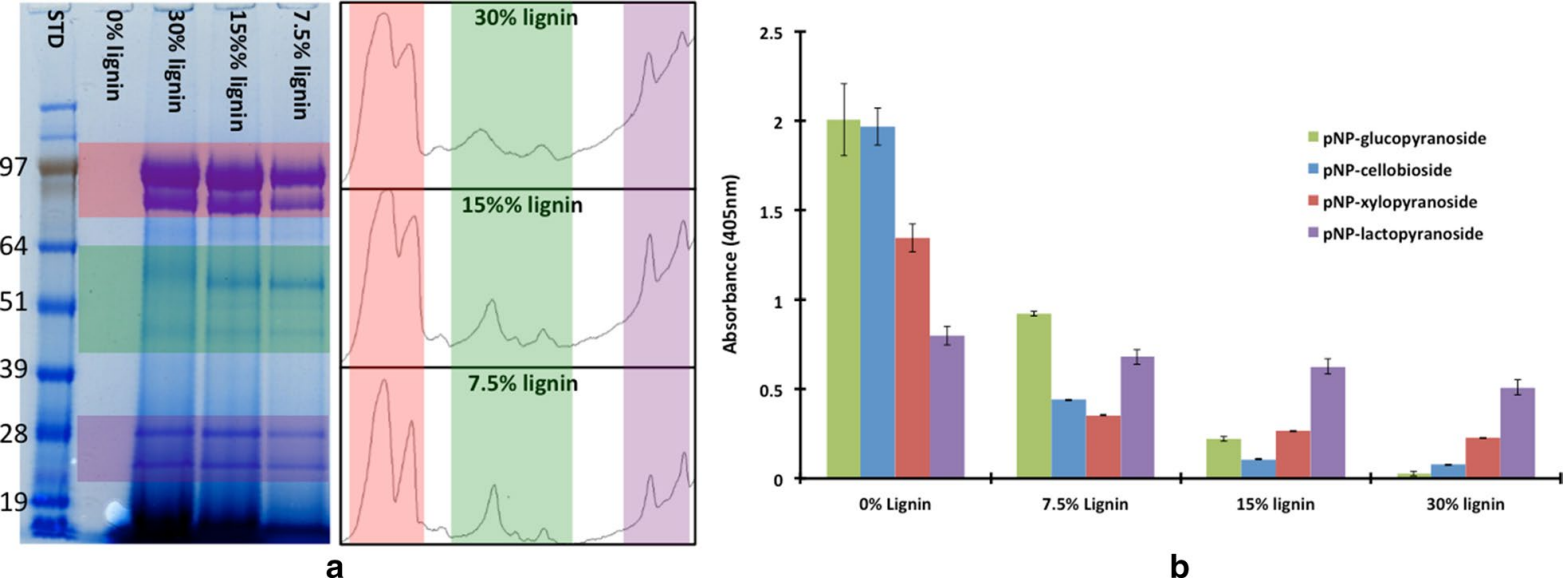
**a** SDS-PAGE gel comparing the proteins bound to lignin at different lignin concentrations with the different molecular regions highlighted (*red* = HMW, *green* = Mid-MW, and *purple* = LMW). **b** *p*NP activities in unbound fractions versus lignin concentration indicating increasing binding as the concentration of lignin is increased

The clear correlation between bound enzyme bands and activity suggests several key questions in understanding enzyme–lignin interactions: (1) which physicochemical properties of the lignin and enzyme drive this interaction and (2) does the binding of these proteins to lignin impact biomass hydrolysis? According to the relationship matrix proposed in Fig. 1, binding of protein to lignin is interdependent on the properties of both components. To begin answering these questions, CTec2 was incubated with 30, 15, 7.5, and 0 % (w/v) lignin while holding the protein concentration constant. Sodium dodecyl sulfate (SDS)-polyacrylamide gel electrophoresis (PAGE) analysis of the bound enzyme shows that the overall protein bound to lignin increases as the concentration of lignin increases (Fig. 4a). The 0 % lignin control indicates that no precipitation of protein occurred. These data also indicate that proteins around ~24, 28, 47, 54, 85, and 97 kDa are primarily adsorbed to lignin.

The evidence of enzymes binding to lignin is further demonstrated in the overall loss in *p*NP-activity (Fig. 4b). There is a large impact from lignin adsorbing on the *p*NPC, *p*NPG, and *p*NPX activities, with an almost 100 % loss of both *p*NPG and *p*NPC activities, and an 83 % loss of *p*NPX activities (Table 2); whereas only 36 % of the *p*NPL loss in activity is associated with these enzymes absorbing to lignin. These results demonstrate that the enzymes associated with *p*NPG, *p*NPC, and *p*NPX activity have the highest affinity for lignin.

**Table 2:** The total percent loss in *p*NP activity due to proteins binding to lignin as lignin concentration is increased

*p*NP substrates	Activity loss in *p*NP activity in the unbound fraction due to different concentrations (%) of lignin		
	7.5 %	15 %	30 %
*p*NP-cellobioside (β-d-glucosidases, endoglucanases)	78	95	96
*p*NP-xylopyranoside (xylanases; β-d-xylobiosidases)	74	80	83
*p*NP-glucopyranoside (β-d-glucosidases)	54	89	99
*p*NP-lactopyranoside (cellobiohydrolases, cellulases)	15	22	36

**Fig. 5:**
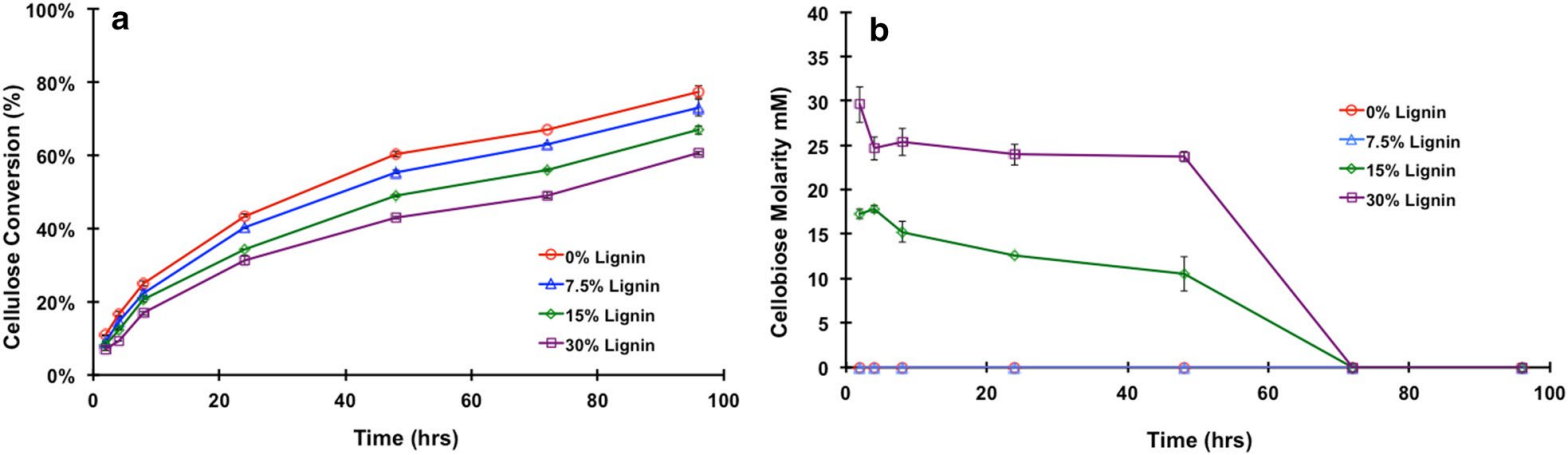
**a** Percent of theoretical glucose conversion by the unbound protein fraction of Cellic CTec2 exposed to different levels of insoluble lignin. **b** Cellobiose concentration in the digestion supernatant

Whereas *p*NP-substrates are simple and easy diagnostic tools in evaluating the activities of enzymes bound to lignin, they do not give insight into how this binding affects glucan conversion in a batch mode. The bound enzymes are not separated from the mixture during batch digestion and could still participate in the hydrolysis of the biomass. To evaluate these activities, the unbound supernatants CTec2 from the lignin-binding experiments were utilized to hydrolyze Avicel. Loadings of supernatants were standardized to 20 mg protein/g of cellulose for all digestions and the changes in the glucose and cellobiose concentrations are shown in Fig. 5a, b, respectively.

From the glucose conversion data in Fig. 5a, we know that the loss of proteins has a significant effect on the overall hydrolysis of cellulose. With respect to the control (0 %), there is a 4, 10, and 16 % decrease in conversion corresponding to a lignin concentration of 7.5, 15, and 30 %, respectively. There is a buildup of cellobiose in the 15 and 30 % lignin samples early in the digestion (Fig. 5b), which is known to inhibit cellulase activity, specifically cellobiohydrolase I [21–24]. Most likely, the elevated cellobiose is due to removal of β-d-glucosidase activity (high molecular weight bands) through binding to lignin. The continued conversion over time (Fig. 5a) and the eventual decrease of cellobiose to undetectable concentrations (Fig. 5b) indicate that either some β-d-glucosidase activity remains in the supernatant or that β-d-glucosidase-type activity from other, unbound cellulases is hydrolyzing the cellobiose. It has been shown that cellobiose in concentrations of 10 mM or greater inhibit the activity of Cel7A [23, 24]. For lignin concentrations of 15 and 30 %, the cellobiose concentration observed is 15 and 30 mM, respectively. The reduction of Avicel hydrolysis by 10 % (for 15 mM cellobiose concentration) and 16 % (for 30 mM cellobiose concentration) can be associated with the buildup of cellobiose due to the lack of β-d-glucosidase in the unbound (supernatant) protein fraction used in the digestions. From these data and data in Fig. 3, it can be determined that Cel7A has little affinity in comparison to the other enzymes found within CTec2. Therefore, in this study more focus was given to the activities of *p*NPX, *p*NPC, and *p*NPG to better understand how the enzymes associated with these activities interact with lignin as process conditions are altered.

### Physiochemical parameters affecting enzyme binding to lignin

To gain insight into the physiochemical mechanisms that play a role in the adsorption of enzymes to lignin, primarily hydrophobicity and electrostatics, we evaluated the influence of pH and salt concentrations on the observed binding at 50 °C for all experiments.

#### pH dependence

**Fig. 6:**
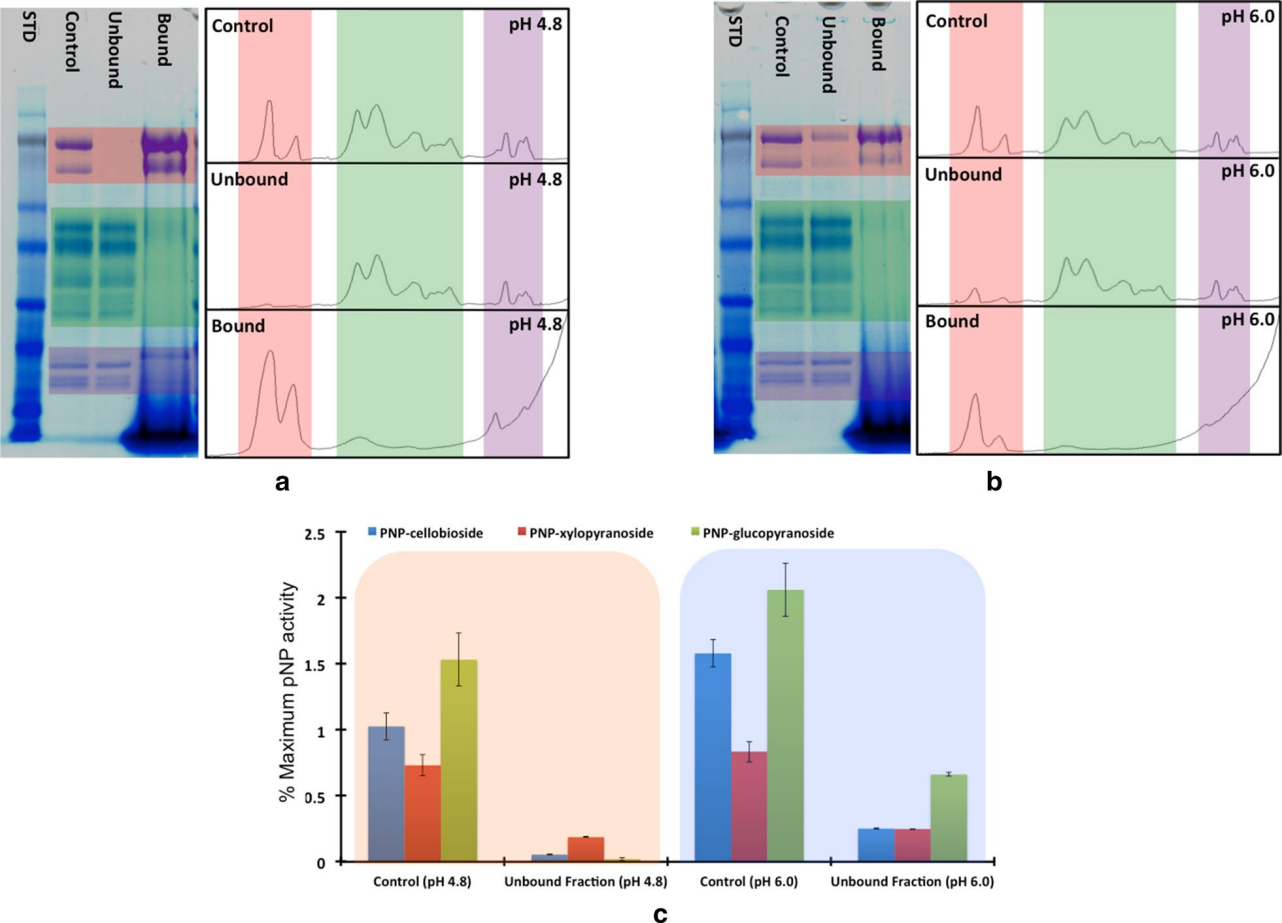
SDS-PAGE and line cuts at **a** pH 4.8 and **b** pH 6.8. **c** Unbound fraction *p*NP activities at pH 4.8 and 6.0

CTec2 was desalted and incubated at pH 4.8 and 6.0 with lignin extracted from corn stover. The supernatant was analyzed by gel electrophoresis and *p*NP activity measurements (Fig. 6a, b). At pH 4.8, the high-molecular-weight bands are almost completely removed from the supernatant presumably by binding to lignin, and a small fraction of the low-molecular-weight bands is bound (Fig. 6a). In contrast, at pH 6.0, these bands are present in the bound fraction, but an overall decrease in the amount of enzyme bound to lignin is apparent, both by the intensity of the bound fraction bands and the presence of light bands in the unbound sample (Fig. 6b). This decrease in the binding affinity of proteins to lignin at pH 6.0 is further demonstrated by the *p*NP activities, where *p*NPG, *p*NPC, and *p*NPX activity loss in the supernatant correlates with the bands binding to lignin (Fig. 6c). The intermediate-molecular-weight bands show little binding at either pH. Table 3 shows an 11, 4, and 31 % decrease in the *p*NP-C, -X, an -G activities of the pH 6.0 unbound compared to the pH 4.8 unbound fractions, signifying less protein binding to lignin at pH 6.0. This difference in the overall loss in *p*NP activity between pH 4.8 and 6.0 suggests electrostatics may play in the interaction between specific enzymes and lignin.

**Table 3:** Percent loss in *p*NP activity due to the proteins being bound to lignin as a function of pH

*p*NP substrates	Activity loss in *p*NP activity in the unbound fraction at different pH values	
	pH 4.8	pH 6.0
*p*NP-cellobioside (β-d-glucosidases, endoglucanases)	95 (%)	84 (%)
*p*NP-xylopyranoside (xylanases; β-d-xylobiosidases)	74 (%)	70 (%)
*p*NP-glucopyranoside (β-d-glucosidases)	99 (%)	68 (%)

Increasing pH increases the negative charge on lignin surfaces, which is hypothesized to increase Coulombic repulsion between most cellulase enzymes and lignin, thereby decreasing enzyme adsorption [25]. Fungal cellulase enzymes have highly varied surface properties, however, and individual enzyme components do not adsorb equally to lignin [17]. The enzymes secreted from cellobiose inducted *Trichoderma reesei*, evaluated here, display a wide range of pI values (pH 5.1–9.1) when separated with isoelectric focusing electrophoresis [26]. To better understand how single enzymes bind to lignin as a function of pH, we analyzed enzymes desorbed from lignin by molecular weight (Fig. 6a). Indeed, we find that for most *T. reesei* proteins (i.e., the acidic species), increased pH leads to decreased enzyme adsorption to lignin, in agreement with results reported previously [27]. We further evaluate enzyme adsorption to lignin under varied salt concentrations. It is generally known that attractive forces dominate repulsive under high salt concentrations, while repulsive interactions dominate attractive forces under low-salt conditions; however, pH effects are important to consider [28]. Since increasing ionic strength shields Coulombic repulsion and attraction, enzyme adsorption to lignin is expected to decrease with increasing salt concentration for basic proteins and increase for acidic proteins, especially at physiological pH (i.e., >6.0). In our work, increasing salt concentration decreases apparent enzyme adsorption to lignin (Fig. 6; Table 4). Indeed, the decrease in adsorption of specific enzymes (xylanases and β-d-glucosidases; the basic enzymes in the secretome) with increasing ionic strength is most pronounced at pH 6 (Table 4). Furthermore, such interactions often depend not only on the overall surface charge, but also on the charge distribution, a factor beyond this study [29]. These results highlight the importance of understanding surface properties for all cellulase components.

**Table 4:** The total percent loss in *p*NP activity due to the proteins being bound to lignin at different pH and ionic strengths of the buffering solution

*p*NP substrates	Activity loss in pNP activity in the unbound fraction at different pH and salt concentrations			
	pH 4.8		pH 6.0	
	50 mM	300 mM	50 mM	300 mM
*p*NP-cellobioside (β-d-glucosidases, endoglucanases)	87 (%)	81 (%)	73 (%)	65 (%)
*p*NP-xylopyranoside (xylanases; β-d-xylobiosidases)	76 (%)	47 (%)	72 (%)	37 (%)

#### Ionic effects

**Fig. 7:**
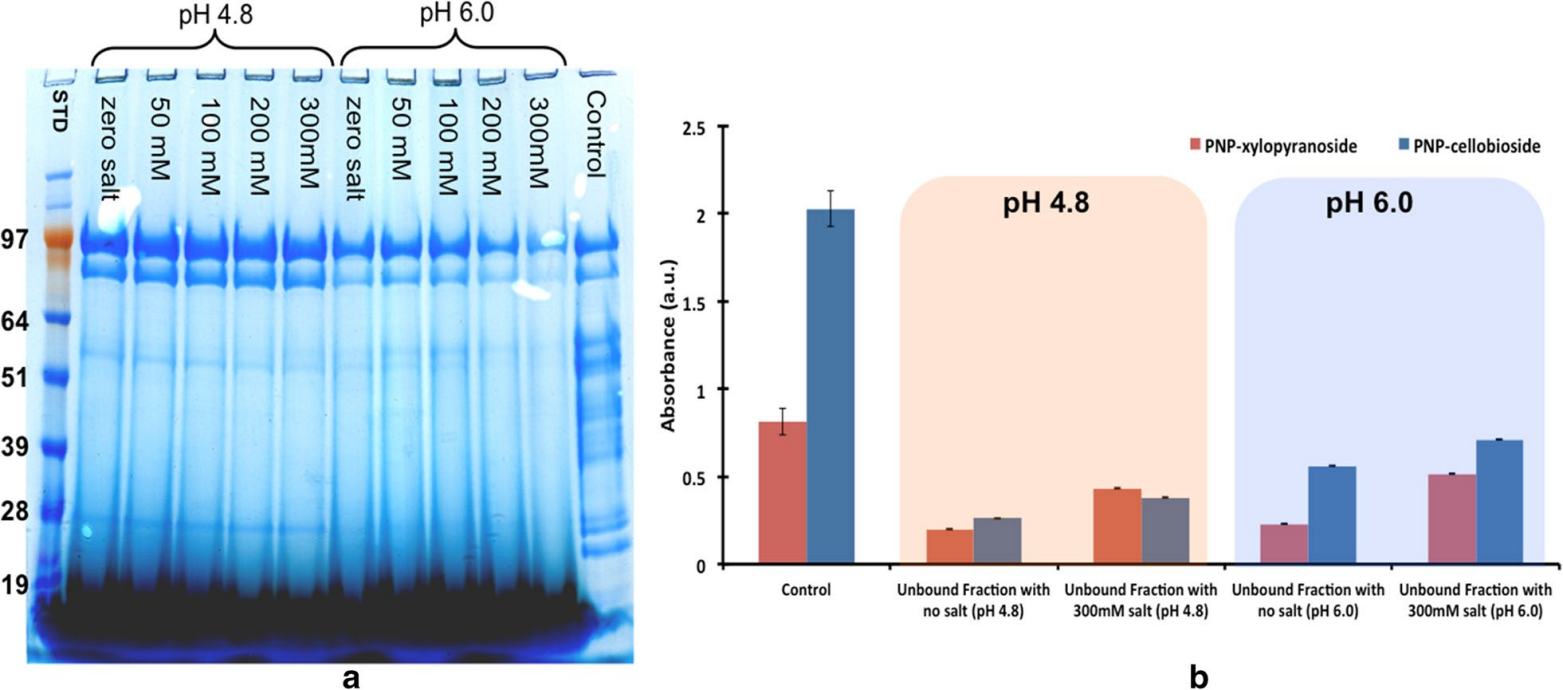
**a** SDS-PAGE comparing the commercial cellulase proteins bound to lignin as a function of pH and NaCl concentration (*lanes* 2–11). *Lane* 1—molecular weight standard. *Lane* 12—enzyme control. **b** Unbound *p*NP activities as a function of binding pH

To study the effect of ionic strength on the overall binding of proteins to lignin, the commercial cellulase product was incubated with lignin at pH 4.8 and 6.0 with five different NaCl concentrations for 1 h. We observed a decrease in the amount of protein binding to lignin as the ionic concentration increases (Fig. 7a), though the effect is much more apparent at pH 6.0 than pH 4.8 due to lower overall protein binding at the higher pH. The *p*NP activities of unbound protein in Fig. 7b also show an increase as the salt concentration increases, indicating less binding at higher ionic strength, especially for *p*NPX activity. Table 4 details the percent difference in *p*NP activity loss due to proteins binding with a more pronounced decrease in the activity loss at higher pH and as the salt concentrations increase.

### Inhibition of enzymes

The experiments described above indicate that removal of the lignin pellet along with the bound enzymes results in a decrease in the catalytic capability of the unbound cellulase mix. Two major questions still remain: (1) are the enzymes bound to lignin still active and if so, can they continue to function in the bound state? (2) Does the presence of soluble low-molecular-weight lignin (SLMWL) impact cellulose digestion?

#### Impact of low-molecular-weight lignin (LMWL) on cellulase activity

Previous gel permeation chromatography (GPC) data indicated a molecular weight distribution for lignin used in this research was between 200 and 10,000 Da. Avicel digestions were carried out under the following four conditions:

1. Control-Avicel = Avicel + CTec2 (red data)
2. Avicel + Lignin = Avicel + CTec2 + lignin (blue data)
3. Avicel + SLMWL = Avicel + CTec2 + soluble LMW lignin (green data)
4. Avicel + Unbound Fraction = Avicel + lignin-depleted CTec2 (purple data points).

**Fig. 8:**
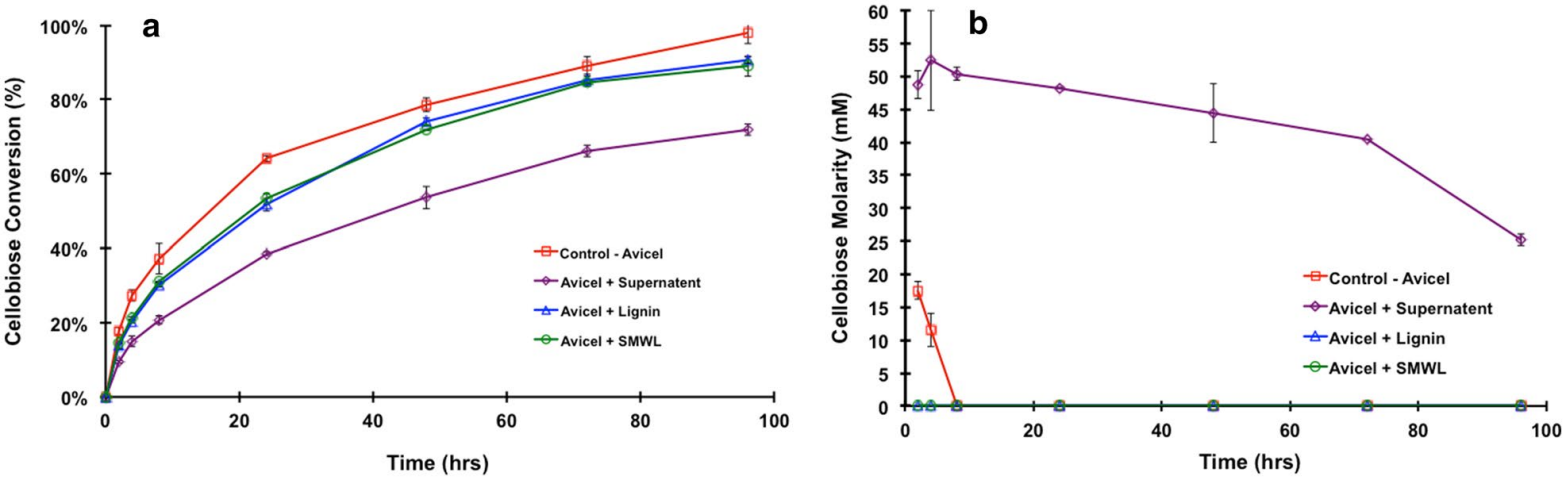
**a** Percent of cellulose conversion. **b** Percent cellobiose concentration

The soluble LMWL (condition 3) is the supernatant from lignin incubation without CTec2. Lignin-depleted CTec2 (condition 4) is the supernatant after binding CTec2 to lignin and removing the lignin and lignin-bound enzymes by centrifugation. Figure 8a, b shows the total glucose conversion and the overall cellobiose concentration over time during the digestion.

Avicel digestion with unbound CTec2 enzymes only (purple data) results in reduced conversion versus digestion of Avicel with complete CTec2 (red data, Fig. 8a). The prolonged production of cellobiose using the lignin-depleted commercial cellulase product suggests that end-product inhibition of cellobiohydrolase I may be the primary cause of the observed productivity loss (Fig. 8b). This concept is consistent with data shown in Fig. 4. From the glucan conversion levels, it can be seen that there is a 26 % difference between the complete CTec2 positive control (red line) and the unbound CTec2 proteins (purple line). This result indicates that the enzymes bound to lignin are important for cellulose hydrolysis. The presence of lignin during the Avicel digestion (blue data) results in only around 10 % decrease in glucan conversion, suggesting that the important enzyme activities missing in the lignin depleted digestion (purple data) are still active when bound to lignin. It is also apparent that the β-d-glucosidase activity, which seems to be especially susceptible to lignin binding, remains active in the presence of lignin as cellobiose does not accumulate (blue data, Fig. 8b). Our previous work suggested that the hydrophobic patches likely responsible for β-d-glucosidases binding to lignin are on the upper surface [17]. We propose that the β-d-glucosidase essentially binds “upside down” to the lignin surface, leaving its active site exposed to soluble cellobiose. As β-d-glucosidases do not have carbohydrate binding domains, this may be a natural mechanism to keep the β-d-glucosidase enzymes in proximity to the cellulases (i.e. bound to lignin) without sterically blocking cellulase accessibility to the cellulose surface. The Avicel + SLMWL (green line) experiment also resulted in a 10 % decrease in the total glucose conversion at 96 h compared to the Avicel control. As there are no insoluble lignins present in this digestion, the inhibition is likely due to soluble LMW lignins inhibiting one or more enzymes. It is possible that this inhibition also accounts for the decrease in conversion when insoluble lignin is present, as the soluble LMW lignin compounds would presumably derive from the lignin fraction. This suggests that the bound enzymes retain close to 100 % of their activity.

### Broader implications

**Fig. 9:**
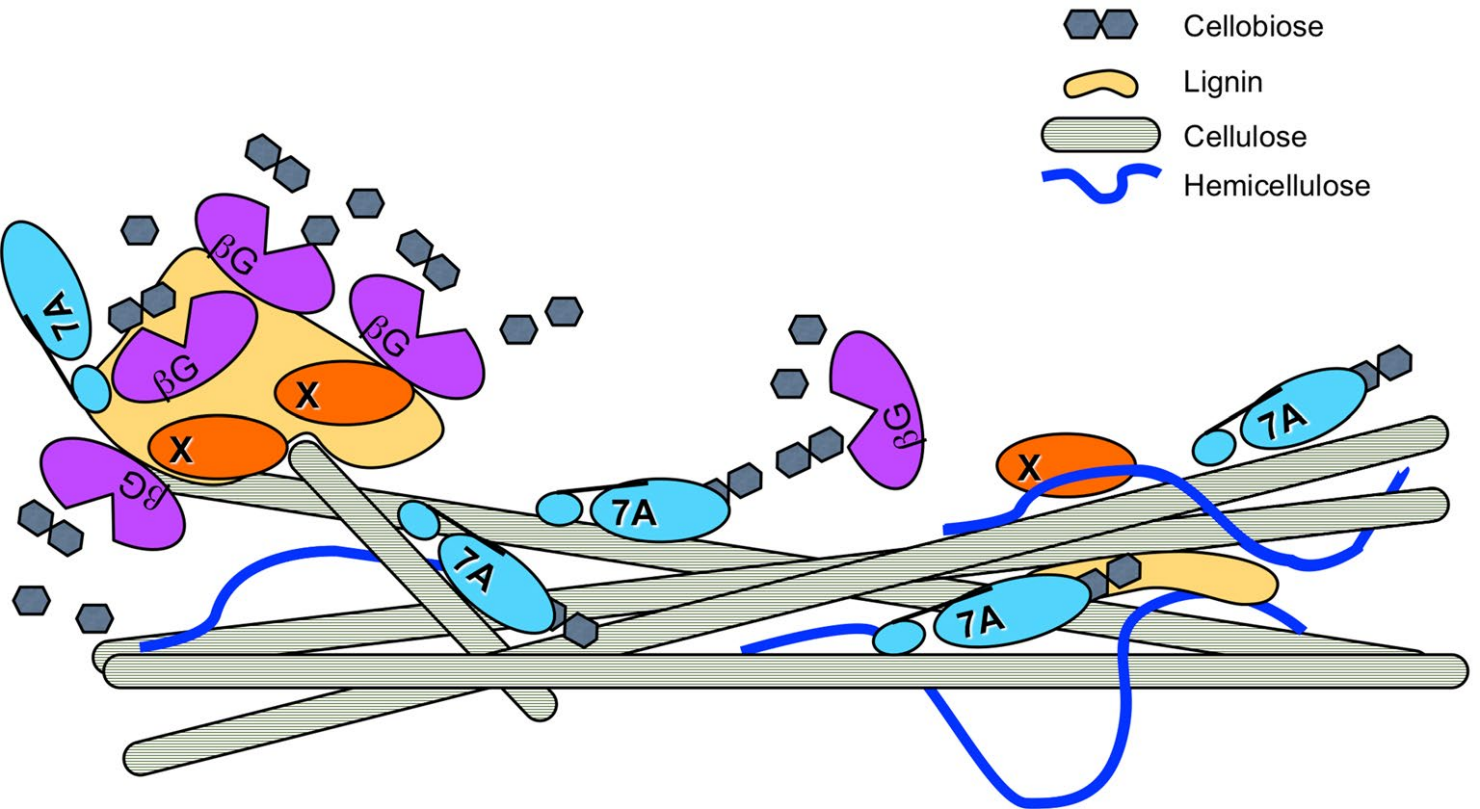
Proposed model of higher lignin-affinity enzymes (β-d-glucosidases and xylanases) displacing CBM-bound cellobiohydrolase from lignin, allowing higher rates of cellulose hydrolysis while retaining functional activity of the bound enzymes

The utility of separating two protein populations, lignin bound and unbound, and their characterization has proven to be a valuable and instructive approach to developing a baseline understanding of the lignin–cellulase adsorption paradigm. Cel7A is thought to account for roughly 40–50 % of the total protein complex secreted in the *T. reesei* system and plays a major role in the processive depolymerization of cellulose. Research efforts to date have led to the current paradigm that Cel7A and Cel7B adsorb to lignin via hydrophobic interactions with the cellulose-binding module (CBM), especially at elevated process temperatures [16, 17, 20]. In contrast to those findings, we are suggesting that for this specific extracted lignin from corn stover, a new paradigm is in play, in which enzymes not containing CBMs appear to have a higher affinity for lignin (as seen in the cartoon in Fig. 9). Moreover, that unbound supernatants have lost nearly all related activities associated with those components These enzymes can displace CBM–lignin bound proteins, as supernatants of mixed cellulase exposed to lignin have lost nearly all related activities associated with those components, yet retain cellulase activity. For example, it appears β-d-glucosidase and some xylanases have a greater affinity for lignin than the β-1-4-exoglucosidases and β-1-4-endoglucosidases when an enzyme mix is used, a finding that may not have been realized if only individual enzymes were compared. The most likely explanation for this observation is that in a mixed enzyme population, higher affinity enzymes can displace lower affinity types. Allowing the individual enzymes to competitively adsorb to the lignin surface demonstrates the known Vroman effect and has led to the hypothesis that it may be possible to predict the relative adsorption characteristics of individual enzymes based on their inherent physiochemical structure as shown in Sammond et al. [17]. In that study, we evaluated the role of hydrophobic interactions causing enzyme binding to lignin and demonstrated a correlation between the affinity of the enzyme toward lignin and its hydrophobic cluster scores [17].

### Specific findings

#### Proteins and activities in bound and unbound fractions

When exposed to insoluble lignin, the unbound fraction of CTec2 becomes more β-d-glucosidase- and xylanase-depleted as the lignin concentration increases, with ~100 % of the β-d-glucosidase and 75 % of the xylanase activity being lost. This is evident from both *p*NP assays (Fig. 4) and Avicel hydrolysis assays (Fig. 5) using CTec2 supernatants after exposure to increasing levels of insoluble lignin. The decreasing levels of *p*NPC, *p*NPG, and *p*NPX activities remaining in the unbound fraction with increasing lignin exposure, as well as increasing cellobiose levels during the Avicel hydrolysis experiment are clear indications that β-d-glucosidase and xylanase activities are preferentially retained with the insoluble lignin. The relatively high levels of cellulase activity remaining in the unbound fraction (*p*NPL and Avicel) after exposure to lignin are clear indications that the majority of the cellulase activity does not bind to lignin, at least when presented as a heterogenous mix of enzyme activities from a commercial cellulase.

Fungal β-d-glucosidases typically have higher molecular weights (~80–100 kDa) than endo- and exo-cellulases (~40–65 kDa) while xylanases are generally in the lower range (~20–30 kDa). This is consistent with both low- and high-molecular-weight bands remaining in the bound fraction along with the β-d-glucosidase (*p*NPG, *p*NPC) and xylanase (*p*NPX) activities while the mid-molecular-weight cellulases and cellulase activity (*p*NPL, Avicel) remain in the soluble fraction. From the biochemical assays, there is high probability the high-molecular-weight bands are associated with β-d-glucosidase enzymes, then mid-MW bands are cellulases, and the low MW enzymes are associated with the xylanase activities.

#### Adsorption driving forces

The pH and salt studies suggest that a combination of hydrophobic and electrostatic interactions impact the adsorption of enzymes to lignin. For β-d-glucosidase, hydrophobicity appears to be the dominant interaction, as increases in salt concentration did not have much impact on binding [17]. At pH 4.8, increasing salt concentration from 50 to 300 mM yielded only a 6 % decrease in the percent loss in *p*NP activity observed. At pH 6.0, the loss in *p*NP activity decreased by 8 %. Neither result is very significant if the primary binding mechanism were governed solely by electrostatics. For the LMW xylanase activities, loss of activity was significantly higher as the salt concentration was raised from 50 to 300 mM. At pH 4.8, the loss in activity was 29 % while at pH 6.0, the loss of activity increased to 35 %. Furthermore, overall binding was lower for the LMW xylanases than for the HMW β-d-glucosidases at both pH conditions. These results indicate a much stronger electrostatic interaction between these low MW xylanases and lignin than between the high MW β-d-glucosidase activities and lignin. This observation is reinforced from our previous work where β-d-glucosidase was predicted to have a much higher hydrophobic interaction potential (i.e., h-patch score) than xylanases [17].

#### Role of β-d-glucosidase and xylanases in terms localized adsorption

It is clear from both *p*NP and Avicel hydrolysis studies that lignin-bound β-d-glucosidase is still active and maintains the ability to hydrolyze cellobiose as demonstrated in Fig. 7. Adsorption of β-d-glucosidase onto lignin may not limit the accessibility of cellobiose to the active site, especially in light of our previous work where the dominant hydrophobic patches were predicted to be primarily on the surface distal to the active site [17]. Further study is required; however, to determine to what extent the bound β-d-glucosidase maintains its ability to effectively hydrolyze cellobiose.

Xylanase activity appears to play no vital role in the digestion of Avicel even though Avicel has trace C5 content; therefore, this work only suggests that certain xylanases (presumably the LMW bands) have an affinity toward lignin. Future work on hemicelluloses from relevant biomass samples may be needed to determine the effects of the xylanases binding to lignin.

## Conclusions

We have shown that several process-relevant enzymes have a high affinity for lignin. These enzymes are most likely β-d-glucosidases and xylanases, suggesting a new paradigm on the interaction of enzymes with lignin where strong hydrophobic (β-d-glucosidase) and electrostatic (xylanases) interactions with lignin can displace CBM–lignin interactions, freeing up cellulases while sequestering still active β-d-glucosidase and xylanase activities. Diagnostic activity and cellulose digestion assays were used to determine the impact of these enzymes binding to lignin. The assays showed that almost 100 % of β-d-glucosidase activity was sequestered on the lignin along with a significant amount of xylanase activity. Furthermore, it was demonstrated with Avicel digestions a majority of the bound β-d-glucosidases remain active. The affinity of these enzymes toward lignin appears to be driven by a combination of hydrophobic and electrostatic forces as demonstrated from the pH and salt experiments.

## Methods

### Lignin purification

#### Steam explosion pretreatment of corn stover

Pretreatment of corn stover was conducted in the National Renewable Energy Laboratory 4-L steam explosion reactor at 180 °C, 1 wt% H_2_SO_4_, for 3 min [30]. The reactor is constructed of Hastelloy C-22 for corrosion resistance. A two-inch thick insulating jacket surrounds the steam jacket and temperature-controlled electrical heating bands that encase all external surfaces of the reactor, limiting heat loss to the environment, and reducing condensation inside the reactor during pretreatment. The pre-warmed reactor was loaded with 500 g of acid-impregnated and pressed corn stover (~43 % solids), sealed with the top ball valve, and steam applied to both the top and bottom of the reactor interior to quickly heat (~5 to 10 s) the biomass to reaction temperature. The timer is started when the reactor contents measured by two thermocouples inside the reactor reach reaction temperature. The bottom ball valve is quickly opened at the desired experimental residence time and the pretreated solids are blown into a nylon HotFill^®^ bag inside a 200-L flash tank. The bag is removed from the flash tank, labeled, sealed, and stored at 4 °C until ready for analysis. This allows collection of all steam and volatile components (furfural and acetic acid) in the slurry for more accurate component mass balance measurements.

#### Lignin extraction method

The pretreated corn stover was extracted with aqueous dioxane utilizing a modified Bjorkman method where the milling and reflux steps (0.1 M HCl at 90 °C) have been eliminated because the samples have already been milled and pretreated under acidic conditions [31]. Approximately 100 g wet weight samples of pretreated solid residues were washed five times with DI water to remove all soluble carbohydrates and by products that may have been generated during the pretreatment process. The washing process involves suspension of the sample solids in 100 mL of DI water and filtered using 90 mm Whatman glass fiber (GF/A) filters in appropriate sized Buchner funnels, then resuspended in DI water for the next round of filtration. The water washed solids were then suspended in 600 mL of a 9:1 (v/v) mixture of dioxane (1,4-dioxane, J.T. Baker) and DI water and extracted for 1 h at 120 °C with intermittent stirring to keep the solid particles suspended. The dioxane extracted solids were then filtered over Buchner funnels equipped with Whatman GF/A glass fiber filters to separate the extracted solid residues from the liquor filtrates. The extracted solids were washed with 500 mL of 95 % ethanol followed by a 500 mL wash with DI water.

The soluble solids within the dioxane:water extraction liquors were concentrated to 50–100 mL utilizing a rotary type evaporator, then precipitated by adding cold DI water (4× the final extract volume), and centrifuged in a GSA rotor at 9000 rpm for 30 min. The precipitated solids were washed 3× with 150 mL DI water and freeze dried for characterization by nuclear magnetic resonance, GPC, and Raman spectroscopy, the results of which indicate minor changes to the lignin molecular weight with increasing pretreatment temperature (data not shown). The yield of lignin extracted with 1:9 dioxane was performed, which ranged from roughly 15 to nearly 40 % of the resident lignin content by weight.

### Enzymes adsorption assays

#### Desalting enzyme preparations

Cellic CTec2 was diluted 1:5 with 25 mM sodium citrate buffer (pH 4.8), passed through a 0.2-μm polyether sulfone (PES) syringe filter, and desalted in 10 mL aliquots using two serial HiPrep 26/10 desalting columns (GE Life Sciences, Piscataway NJ) equilibrated in the same buffer. Protein-containing fractions were pooled and protein concentration determined using the bicinchoninic acid protein assay (Pierce Rockford, IL). Enzyme samples were desalted less than 2 days before use, with fresh material being generated for each experiment as desalted commercial enzymes tend to precipitate within a few days.

#### Lignin binding

For binding studies, 300 μg of desalted protein was incubated with 6 mg of lignin, corresponding to a process loading of ~30 mg protein/g cellulose for a theoretical biomass containing 30 % lignin and 50 % cellulose. The protein/lignin combination was incubated at room temperature for 60 min in 25 mM sodium citrate buffer at pH 4.8 unless otherwise stated. After the incubation, the lignin was centrifuged at 20,000×*g* for 5 min and the supernatant containing the unbound protein was collected while the lignin pellet was washed an additional four times with citrate buffer.

#### SDS-PAGE gel assays

Pre-cast 4–12 % SDS-PAGE gels (Life Technologies, Carlsbad, CA) were used to visualize proteins bound and unbound to lignin extracted from corn stover. All gels were run at 200 V constant for 50 min in 3-(*N*-morpholino)propanesulfonic acid (MOPS)-SDS buffer. The desalted starting enzyme, supernatant containing unbound proteins, and the lignin pellet containing protein bound to insoluble lignin were diluted with 4×LDS sample buffer (3:1 sample:buffer) and held at 70 °C for 10 min in preparation for SDS-PAGE.

#### *para*NP-assays

A variety of *p*NP substrates (Sigma-Aldrich, St. Louis, MO) were used to determine the loss of enzymatic activity of commercial enzyme preparations in the presence of lignin. Substrates used were *p*NP-β-d-lactopyranoside, *p*NP-β-d-cellobioside, *p*NP-β-d-glucopyranoside, and *p*NP-β-d-xylopyranoside. The 2.0-mM final *p*NP assay concentration was prepared from a 10.0 mM working solution in 25 mM sodium citrate buffer at pH 4.8.

Desalted commercial enzyme preparations were used at a concentration of 50 μg/mL in a 2-mM *p*NP solution. The *p*NPX and *p*NPL assays were incubated at 45 °C for 30 min. The *p*NPC and *p*NPG were incubated at room temperature for 5 min due to the high activity of enzymes on these substrates. After incubation, the reaction was stopped and color developed by the addition of a twofold volume of 1.0 M sodium carbonate. Absorbance at 405 nm was measured in a spectrophotometer.

### Comparative cellulose digestibility

Enzymatic digestions were carried out in 2.0 mL glass screw-cap high-performance liquid chromatography vials at 1 % (w/v) solids loading at 45 °C while rotating end-over-end at 12 rpm for 96 h. Commercial enzymes were added at a level of 20 mg protein per gram of cellulose. No additional accessory enzymes were added. The total volume of the saccharification slurries after adding enzyme and 50 mM citrate buffer was 2.0 mL. To determine the progress of glucan conversion, 100 μL aliquots of the well-mixed slurries were taken at 2, 4, 8, 24, 48, 72, and 96 h. The samples were immediately diluted with 900 μL of DI water, and the enzymes were inactivated by heating at 95 °C for 12 min. Samples were filtered through Pall Acrodisc nylon 0.2 µm syringe filters (Pall, Port Washington, NY) and refrigerated until HPLC analysis on an Agilent 1100 using a 300 mm × 7.8 mm BioRad Aminex HPX87H ion exclusion column maintained at 55 °C. The mobile phase was 0.01 N sulfuric acid at a flow rate of 0.6 mL/min. The sample injection volume was 20 µL and the run time 25 min. The glucan conversion was calculated by adding the total glucose and cellobiose yields (both glucose and cellobiose were converted to glucan equivalent) for each hydrolysis time point.

## Abbreviations

BCA: bicinchoninic acid
BSA: bovine serum albumin
CBM: carbohydrate-binding module
EDAX: energy-dispersive X-ray spectroscopy
GPC: gel permeation chromatography
HPLC: high-performance liquid chromatography
LDS: lithium dodecyl sulfate
LMWL: low-molecular-weight lignin
MOPS: 3-(*N*-morpholino)propanesulfonic acid
NIST: National Institute of Standards and Technology
NMR: nuclear magnetic resonance
NREL: National Renewable Energy Laboratory
PAGE: polyacrylamide gel electrophoresis
PES: polyether sulfone
*p*NP: *para*-nitrophenol
*p*NPL: *para*-nitrophenyl-β-d-lactopyranoside
*p*NPC: *para*-nitrophenyl-β-d-cellobioside
*p*NPX: *para*-nitrophenyl-β-d-xylopyranoside
*p*NPG: *para*-nitrophenyl-β-d-glucopyranoside (*p*NPG)
SDS: sodium dodecyl sulfate
